# Effectiveness of antidepressants for treatment of idiopathic orofacial pain

**DOI:** 10.1186/1129-2377-14-S1-P47

**Published:** 2013-02-21

**Authors:** M Ikawa, M Ikawa

**Affiliations:** 1 Shimizu Municipal Hospital, Japan; 2 Shimizu Municipal Hospital, Dept. of Oral Surgery, Japan

## Aims

To determine the efficacy of antidepressants for treating idiopathic orofacial pain fulfilling the International Classification of Headache Disorders 2nd edition (ICHD-II) criteria of persistent idiopathic facial pain (13.18.4; IFP) or burning mouth syndrome (13.18.5; BMS).

## Materials and methods

Participants comprised 195 outpatients who attended our orofacial pain liaison clinic between January 1, 2009 and December 31, 2011 and were diagnosed with IFP or BMS. IFP was diagnosed in 124 patients (17 men, 107 women; atypical facial pain, n=16; atypical odontalgia, n=108), with a mean age of 54.8 ±14.4 years and a mean duration of illness of 50.7 ± 62.8 months. BMS was diagnosed in 71 patients (8 men, 63 women; so-called BMS, n=28; glossodynia (pain limited to tongue), n=43), with a mean age of 67.2 ± 10.8 years and a mean duration of illness of 23.1 ± 27.3 months. Patients with mental disorders, including major depressive disorders, who were currently taking antipsychotic agents were excluded. All participants were treated with antidepressants, with first-line therapy comprising amitriptyline or another tricyclic antidepressant (TCA). If a TCA alone proved insufficient for pain control, one of the following agents was added to the TCA for combination therapy, in this order: risperidone; sodium valproate; or lithium. These other antidepressants were also used if the patient could not use TCAs due to underlying disease. Outcomes were assessed as: -effective, pain disappeared or no pain was experienced for 90% of each week; -moderately effective, pain improved, but did not disappear; -no effect, no change; and dropped out, including referral due to difficulty making visits because of the distance.

## Results

For IFP, treatment was effective for 91 patients (73.4%), moderately effective, 3.2%, no effect, 0%, dropped out, 23.4%. For BMS, treatment was effective for 56 patients (78.9%), moderately effective, 4.2%, no effect, 1.4%, dropped out, 15.5%.

## Discussion

Use of antidepressants can be helpful in the treatment of idiopathic orofacial pain such as IFP or BMS.
